# Biomass-Derived Porous Carbon with a Good Balance between High Specific Surface Area and Mesopore Volume for Supercapacitors

**DOI:** 10.3390/nano12213804

**Published:** 2022-10-28

**Authors:** Yanbo Wang, Yiqing Chen, Hongwei Zhao, Lixiang Li, Dongying Ju, Cunjing Wang, Baigang An

**Affiliations:** 1 Key Laboratory of Energy Materials and Electrochemistry Research Liaoning Province, School of Chemical Engineering, University of Science and Technology Liaoning, Anshan 114051, China; 2 School of Chemistry and Materials Engineering, Xinxiang University, Xinxiang 453003, China; 3 State Key Laboratory of Metal Material for Marine Equipment and Application, Anshan 114009, China

**Keywords:** biomass, porous carbon, supercapacitors, mesopore–micropore, salt template

## Abstract

Porous carbon has been one desirable electrode material for supercapacitors, but it is still a challenge to balance the appropriate mesopore volume and a high specific surface area (SSA). Herein, a good balance between a high SSA and mesopore volume in biomass-derived porous carbon is realized by precarbonization of wheat husk under air atmosphere via a chloride salt sealing technique and successive KOH activation. Due to the role of molten salt generating mesopores in the precarbonized product, which can further serve as the active sites for the KOH activation to form micropores in the final carbon material, the mesopore–micropore structure of the porous carbon can be tuned by changing the precarbonization temperature. The appropriate amount of mesopores can provide more expressways for ion transfer to accelerate the transport kinetics of diffusion-controlled processes in the micropores. A high SSA can supply abundant sites for charge storage. Therefore, the porous carbon with a good balance between the SSA and mesopores exhibits a specific gravimetric capacitance of 402 F g^−1^ at 1.0 A g^−1^ in a three-electrode system. In a two-electrode symmetrical supercapacitor, the biomass-derived porous carbon also delivers a high specific gravimetric capacitance of 346 F g^−1^ at 1.0 A g^−1^ and a good cycling stability, retaining 98.59% of the initial capacitance after 30,000 cycles at 5.0 A^−1^. This work has fundamental merits for enhancing the electrochemical performance of the biomass-derived porous carbon by optimizing the SSA and pore structures.

## 1. Introduction

Rechargeable battery technologies are urgently needed to tackle the current energy and environmental concerns of modern society [1,2,3]. As typical electrochemical energy storage and conversion devices, supercapacitors have shown extraordinary promise due to their high power density and outstanding durability [4,5,6,7]. Porous carbons with attractive advantages such as a high specific surface area (SSA), high conductivity, and outstanding chemical stability have been identified as ideal electrode materials for supercapacitors [8,9,10,11,12,13].

Porous carbons are generally obtained with coals, cokes, and pitches or biomass as precursors. In the past few decades, biomass has become a rapidly developed and widely used precursor to fabricate porous carbon electrode materials due to its advantages such as the abundance of raw materials, low cost, and renewability [14,15,16,17,18,19]. For carbon-based supercapacitors, the energy storage involves charge accumulation and separation at the interface of the electrolyte and electrode material during charge and discharge [20,21,22,23]. Based on this mechanism, the specific capacitance of a supercapacitor mainly depends on the SSA of the electrode materials. Therefore, enhancing the SSA of porous carbon materials is the most promising strategy to improve the capacitance performance of supercapacitors [24,25,26]. Although abundant micropores can increase the SSA of the electrode materials and micropores of a size similar to that of the ions can enhance the capacitance [27], the slow ion transport in the long narrow micropores may lead to an inferior rate performance of the supercapacitors [27]. To make a large SSA fully accessible, introducing mesopores is considered as an effective method since the mesopores can support more fast transport ways for ions [28,29,30]. 

Template methods, physical activation (activation with O_2_, CO_2_, or plasma treatment), and chemical activation (activation with KOH, ZnCl_2_, etc.) have been successfully employed to fabricate porous carbons with mesopores, micropores, or hierarchical pores [28,29,30,31]. These porous carbon electrodes improved the electrochemical performance of the supercapacitors to a great extent. However, the relatively low energy density and inferior rate performance still significantly limit their future practical applications. Although a high SSA can provide abundant sites for ion storage and a large mesoporous volume is convenient for the ease of ion transportation, how to balance the high SSA and appropriate mesopore/micropore volume is still a challenge. Recent research has developed new ways to balance the high SSA and mesopore volume of porous carbons, which are useful for good performance in supercapacitors [32,33,34]. Chemical activation of carbonaceous matter adopting KOH as the activating agent is widely used, and the porosity of such carbons depends on the nature of the precursor and activation conditions [35]. Subjecting the precursor to an initial carbonization step before activation can increase the carbon content and reduce the O/C ratio in the material, meaning that fewer oxidizing gases are released upon activation, leading to more controlled activation and the improvement of the porosity of the carbons [35,36,37]. In this technique, biomass is a preferred choice for activated carbons due to being readily available, renewable, and essentially offering a “carbon neutral” route to porous carbons [36,37]. 

Producing value-added carbon electrode materials using wheat husk is a good way to reuse agricultural waste; however, precarbonizing wheat husk in N_2_ atmosphere and further activating the precarbonized carbon with KOH often generate abundant micropores in the carbon material with slow ion transportation kinetics [31,32,33]. To overcome this critical problem, taking into account the template and etching role of molten salt and oxygen on porous carbon produced in air using the salt sealing technique [19,20,21], we developed a facile strategy to construct porous carbon with a good balance between a high SSA and mesopore volume by precarbonization of wheat husk in air and successive KOH activation. The precarbonization process employs a mixture of KCl and NaCl as the salt template to generate mesopores in the carbon framework; meanwhile, the mixed salt can act as a shielding agent to prevent the carbon framework from oxidation in air at a high temperature. The mesopores in the carbon framework can provide active sites for successive KOH activation to produce a large amount of micropores. Benefiting from the synergistic pore-generating effect of molten salt and KOH, the obtained porous carbon exhibits a hierarchical pore structure with a good balance between a high SSA and mesopore volume. The high SSA provides a sufficient electrode/electrolyte interface for charge accumulation, and the high mesopore volume supplies more expressways for fast ion transfer. The biomass-derived porous carbon exhibits a high specific gravimetric capacitance of 402 F g^−1^ at 1.0 A g^−1^ in a three-electrode system. In a two-electrode symmetrical supercapacitor, the biomass-derived porous carbon also delivers an excellent specific gravimetric capacitance of 346 F g^−1^ at 1.0 A g^−1^ and a good cycling stability with a retention of 98.59% after 30,000 cycles at 5.0 A^−1^. 

## 2. Material and Methods

### 2.1. Synthesis of Porous Carbon

Firstly, the mixture of KCl and NaCl in a mass ratio of 1:1 was ground. The wheat husks were washed with distilled water and dried at 60 °C in an oven. Then, 4.0 g of wheat husk was mixed with 16.0 g of a KCl and NaCl mixture in a 50 mL porcelain crucible, precarbonized at 700 °C, 800, and 900 °C for 1 h in a muffle furnace in air atmosphere. The obtained precarbonized product was washed with distilled water and dried at 80 °C, mixed with KOH at a weight ratio of 1:2 (carbon/KOH) in an agate mortar. The precarbonized product was further activated by KOH in a tubular furnace at 800 °C for 1 h under nitrogen atmosphere to obtain the porous carbon. The porous carbons as prepared were washed with 3.0 M HCl solution and distilled water until the pH of the washing effluent reached 6–7, then the filtered product was dried at 80 °C in an oven overnight. According to the carbonization temperature, the porous carbons derived from wheat husk were named HPC-700, HPC-800, and HPC-900, respectively. In addition, the precarbonized products at 700 °C, 800, and 900 °C in a muffle furnace in air atmosphere for 1 h were named MHPC-700, MHPC-800, and MHPC-900. Wheat husk was also precarbonized at 700 °C in nitrogen atmosphere without molten salt for 1 h, and the precarbonized product was named as NHPC-700. The carbon yield of the different synthesis processes is summarized in Table 1.

### 2.2. Material Characterizations

Field emission scanning electron microscopy (SEM, FEI Quanta FEG 250, Hillsboro, OR, USA), transmission electron microscopy (TEM, JEM-2100, JEOL USA, Peabody, MA, USA), X-ray diffraction (XRD; Bruker D8 Advance, Billerica, MA, USA) equipped with Cu Kα radiation (λ = 1.5418 Å), X-ray photoelectron spectroscopy (XPS) using a Perkin-Elmer PHI-5700 ESCA System (Waltham, MA, United States) multifunctional photoelectron spectrometer with Al Kα radiation (1486.6 eV), and a NOVA2200e physisorption analyzer were used to examine the morphology, microstructure, crystallographic structure, surface chemical species, and porous texture of the obtained porous carbons. In addition, the specific surface area and the pore size distribution of the carbon materials were calculated by the Brunauer–Emmett–Teller (BET) equation from the nitrogen adsorption data in the relative pressure (*P/P_0_*) of 0.03~0.30 and the nonlocal density functional theory (NLDFT) equilibrium model for cylinder/slit pores from N_2_ sorption data. The total pore volume (*V*_total_) was determined at a relative pressure *p/p*_0_ = 0.990 and the micropore volume (*V*_micro_) using the t-plot method. 

### 2.3. Electrochemical Measurements

Electrodes were prepared by painting a paste containing the porous carbon, carbon black, and polytetrafluoroethylene (PTFE) in a weight ratio of 80:15:5 onto a current collector of stainless steel mesh; the mass loading of the electrode materials was 3 mg cm^−2^. The electrochemical tests of the individual electrode including cyclic voltammetry (CV), galvanostatic charge/discharge (GCD), and electrochemical impedance spectroscopy (EIS) were measured on a CHI660C electrochemical work station in a 1 mol L^−1^ H_2_SO_4_ solution in a three-electrode system. The porous carbon electrodes, a platinum foil, and a Ag/AgCl electrode served as the working, counter, and reference electrodes, respectively. The CV at scan rates from 10–100 mV s^−1^ and GCD tests at current densities ranging from 1.0 to 20.0 A g^−1^ were recorded between 0 and 1 V (vs. Ag/AgCl). EIS was obtained in the frequency range from 100 kHz to 0.1 Hz with a 5 mV AC voltage amplitude. The symmetric supercapacitors were assembled with a glassy fibrous separator and tested in a 1 mol L^−1^ H_2_SO_4_ solution with a LAND CT2001A instrument. 

The gravimetric specific capacitance of a single electrode, *C*_G_ (F g^−1^), was calculated from the discharge curve according to the equation

(1) $$C_{G}=\frac{I}{m\times \left(\Delta U/\Delta t\right)}$$

where *I* is the constant charge/discharge current, Δ*t* is the discharge time, Δ*U* is the potential window during the discharge process, and *m* is the mass of the active materials in a single electrode.

The gravimetric specific capacitance of a single electrode in a two-electrode system, *C*_sG_ (F g^−1^), was calculated from the discharge curve based on the equation.

(2) $$C_{\mathrm{sG}}=4\times \frac{I}{m\times \left(\Delta U/\Delta t\right)}$$

where *m* is the total mass of active materials in the two electrodes. The gravimetric energy density of the device *E*_G_ (Wh kg^−1^) was estimated by using the following equation:

(3) $$E_{G}=\frac{1}{28.8}C_{sG}\Delta U^{2}$$

and the gravimetric power density of the device *P*_G_ (W kg^−1^) was calculated according to the equation

(4) $$P_{G}=\frac{3600E_{G}\;}{\Delta t}$$

## 3. Results and Discussion

**Scheme 1:**
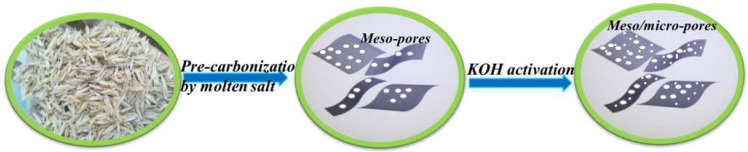
Schematic synthesis process for the HPC.

Assuming that molten salt can penetrate into the carbon skeleton to serve as a “cutting” reagent or template [19,20,21], as illustrated in Scheme 1, the wheat husk was first precarbonized under air atmosphere by a simple salt sealing technique employing low-cost and non-toxic mixed salt of KCl and NaCl as a dual function agent to prevent the carbon structures from oxidation at 700~900 °C above the melting temperature of the salt mixture of NaCl and KCl (669 °C for the equal mass mixture [20]) to build mesopore structures during the precarbonization process. Then, the mesopores generated in the carbon materials further served as active sites for the successive KOH activation to produce a large amount of micropores. The morphologies and microstructure of the carbon samples were examined by SEM and TEM. 

**Figure 1:**
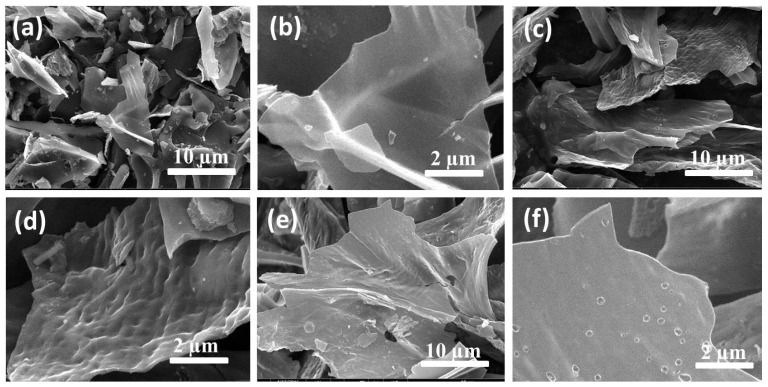
SEM images of (**a**,**b**) HPC-700, (**c**,**d**) HPC-800, and (**e**,**f**) HPC-900.

**Figure 2:**
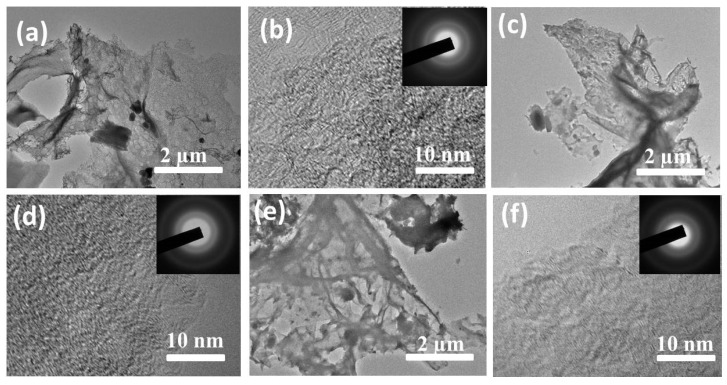
TEM images of (**a**,**b**) HPC-700, (**c**,**d**) HPC-800, and (**e**,**f**) HPC-900 (the inset is the corresponding SAED images).

**Figure 3:**
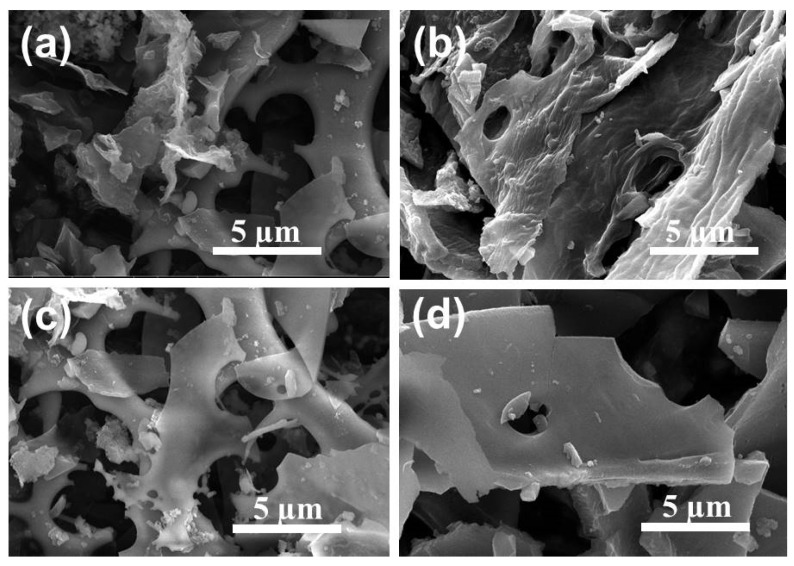
SEM images of (**a**) MHPC-700, (**b**) MHPC-800, (**c**) MHPC-900, and (**d**) NHPC-700.

Figure 1a,b display the SEM images of HPC-700, in which irregular carbon sheets are observed and the surface of the carbon sheets looks very smooth. The surface of the carbon sheets of HPC-800 (Figure 1c,d) looks rougher than HPC-700, and obvious macropores are observed in the carbon sheets of HPC-900 (Figure 1e,f). The changes of the sheet morphologies of HPC-800 and HPC-900 can be ascribed to the stronger etching effect of the molten salt at a higher temperature. For the mesoporous–microporous carbon electrode materials, the sheet structure can reduce the ion transport distance in the carbon electrodes and accelerate the ion transfer kinetics in the micropores limited by diffusion control [19,20,21]. The sheet carbon structure was further confirmed by low-resolution TEM images of the three samples shown in Figure 2a,c,e. Consistent with the SEM results, the surface of HPC-700 looks smoother and transparent. In addition, obvious mesopores in HPC-700 are observed in Figure 2a. The corresponding high-resolution TEM images of the samples in Figure 2b,d,f show that the carbon sheets consist of multilayer discontinuous graphite stripes with a low graphitization degree, consistent with the selected area electron diffraction (SAED) images with the diffuse rings in the insets. To investigate the formation process of the sheet morphologies of the carbon samples, Figure 3a–c show the SEM images of the carbon samples MHPC-700, MHPC-800, and MHPC-900 precarbonized in molten salt. By comparison with the SEM image of the carbon sample NHPC-700 precarbonized in nitrogen atmosphere without molten salt (Figure 3d), it can be observed that the obvious sheet morphology dominates in MHPC-700, while a thick carbon block is prominent in NHPC-700, demonstrating that a sheet structure of the carbon samples HPC-700, HPC-800, and HPC-900 was formed during the precarbonization process in molten salt. In this precarbonization process, wheat husk firstly experiences a steady transition from sp^3^C-X (X: e.g., C, O, H) bonds to the aromatic sp^2^ C-C bonds to form the carbon skeleton; when the temperature is above the melting temperature of the salt mixture of NaCl and KCl, the molten salt diffusing into the carbon skeleton functions as a “cutting” reagent to prohibit the stacking of the sp^2^ coordinated carbon layers along the C-axis due to the van der Waals force [20]; meanwhile, the high energy Cl^−^ ions in the molten salt media continue to etch the carbon structures, leading to the final formation of carbon sheets with multilayer discontinuous graphite stripes [20].

**Figure 4:**
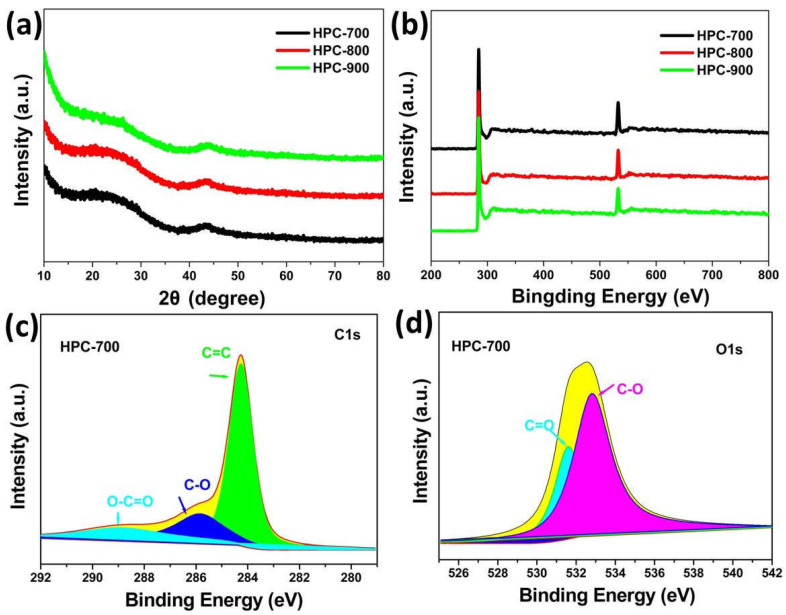
(**a**) XRD patterns and (**b**) XPS survey spectra of HPC-700, HPC-800, and HPC-900; (**c**) C1s and (**d**) O1s spectra of HPC-700.

The XRD patterns of the samples are shown in Figure 4a. The broad and weak peaks at about ~25° and ~44° attributed to the (002) and (100) planes of graphite further reveal the low graphitization degree of the three carbon samples [36,37]. To analyze the surface composition of the samples, the XP survey spectra of the carbon samples are shown in Figure 4b. In the XP spectra, two peaks at binding energy of 284.6 and 532.4 eV were assigned to C1s and O1s. The content of carbon and oxygen was evaluated to be HPC-700 (89.34 and 10.66 at%), HPC-800 (89.98 and 10.02 at%), and HPC-900 (90.56 and 9.44 at%). The high-resolution C1s and O1s spectra of HPC-700 (Figure 4c,d) show three peaks at 284.5, 285.8, and 287.8 eV, attributed to C=C, C–O, and C=O bonds, and two peaks at 532.8 and 531.6 eV, attributed to C–O and C=O bonds. These functional groups can introduce pseudocapacitance to the carbon electrodes during the charge/discharge process [19,20,21].

**Figure 5:**
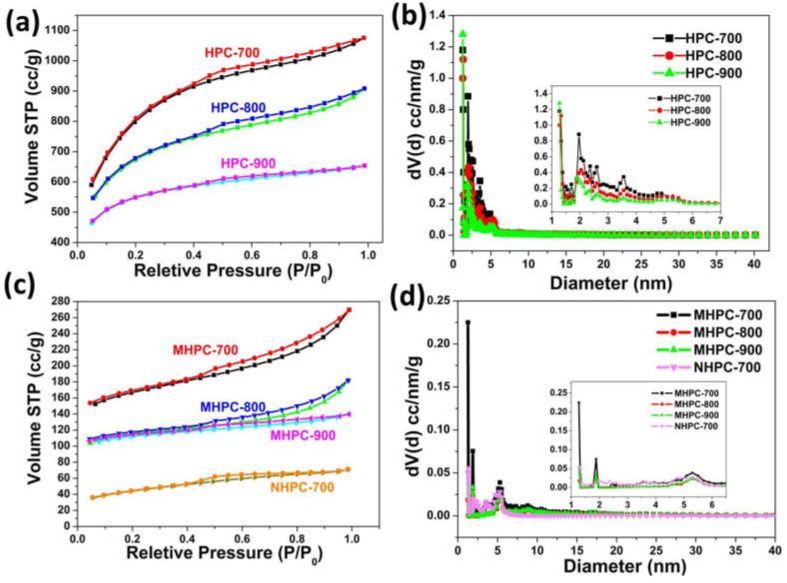
(**a**) N_2_ adsorption/desorption isotherms and (**b**) PSDs of HPC-700, HPC-800, and HPC-900; (**c**) N_2_ adsorption/desorption isotherms and (**d**) PSDs of MHPC-700, MHPC-800 MHPC-900, and NHPC-700.

The pore structures of the samples were further investigated by nitrogen adsorption-desorption measurements at 77 K; the results are shown in Figure 5a. The typical hysteresis loops at *p*/*p*_0_ > 0.4 and high nitrogen uptake at low relative pressure indicate the mesoporous feature and a large amount of micropores. By comparison, HPC-700 has the highest total nitrogen uptake and the biggest hysteresis loop, indicating the largest SSA and mesopore volume. The detailed data in Table 2 suggest that HPC-700 has the highest SSA, *V*_total_, *V*_micro_, and *V*_meso_, possessing a good balance between high SSA and *V*_meso_, which is an advantage for improving the electrochemical performance of the supercapacitors. Figure 5b shows the pore size distribution (PSD) curves of samples based on a nonlocal density functional theory (NDFT) model. No vast differences in the pore size distribution were observed among these samples. These samples all show distributed pore sizes centered at 1.3~6 nm and contain many hierarchical mesopores–micropores. 

As for the formation process of such porous carbon structures with a high SSA and *V*_meso_, it is assumed that the synergistic pore-generating effect of the molten salt and KOH is crucial. As reported in the literature, molten salt plays important template and etching roles in generating mesopores in porous carbon, and traces of oxygen penetrated into the system can generate micropores [19,20,21]. By comparison, the obviously higher total nitrogen uptake of MHPC-700, MHPC-800, and MHPC-900 than that of NHPC-700 and the big hysteresis loops in their nitrogen adsorption–desorption isotherms (Figure 5c) confirm the role of molten salt and the trace of oxygen in generating mesopores and micropores. The hysteresis loops of NHPC-700 were ascribed to inorganic salts in the wheat husk. The detailed data in Table 2 further suggest that NHPC-900 has the lowest SSA and MHPC-700 has a higher SSA and mesopore volume than those of MHPC-800 and MHPC-900. Moreover, their PSDs in Figure 5d also reveal their prominent mesopore features. These data further suggest that, when the temperature of precarbonization is 700 °C, the mesopore amount generated in the carbon skeleton is just suitable for the successive KOH activation, thus leading to the highest SSA of HPC-700. With the temperature of precarbonization increased to 800 and 900 °C, a part of the mesopores collapses, resulting in the obvious decrease in the *V*_meso_ and SSA of MHPC-800, MHPC-900, HPC-800, and HPC-900.

**Figure 6:**
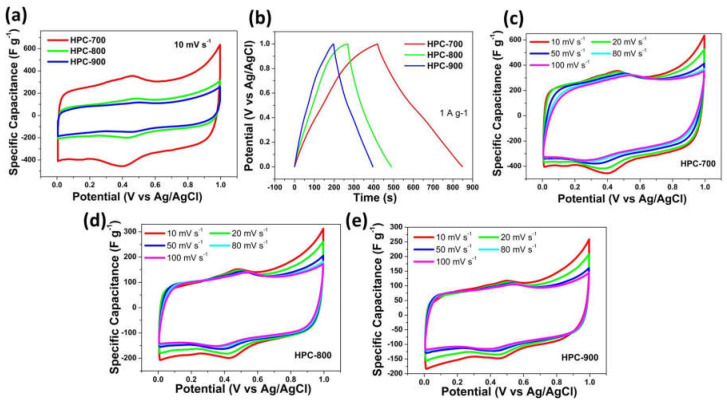
(**a**) CV curves at 10 mV s^−1^ and (**b**) GCD curves at 1.0 A g^−1^ of HPC-700, HPC-800, and HPC-900; CV curves of HPC-700 (**c**), HPC-800 (**d**), and HPC-900 (**e**) at different scanning rates.

Considering the high SSA and mesopore volume suitable for charge accumulation and rapid ion transfer, these carbon samples are expected to exhibit excellent electrochemical performance for supercapacitors. Figure 6a shows the CV at a scan rate of 10 mV s^−1^ with a quasi-rectangular shape and the broad redox peaks around 0.2~0.6 V, indicating an ideal electrical double-layer capacitance of the porous carbon electrode materials [19]. Compared with the other samples, HPC-700 displays the largest enclosed CV curve area, indicating the highest specific capacitance. The GCD curves of the samples at 1.0 A g^−1^ are further shown in Figure 6b. HPC-700 shows a longer discharge time, signifying a higher specific capacitance of 402 F g^−1^ than that of HPC-800 (210 F g^−1^) and HPC-900 (199 F g^−1^). The excellent specific capacitance of HPC-700 results from the good balance between its large accessible SSA, providing a more efficient electrode–electrolyte interface and the rational *V*_meso_ supplying more rapid ion transportation pathways [38]. Based on the advantage of this balance, the wheat-husk-derived carbon HPC-700 exhibits a remarkable capacitance compared with the other biomass-derived carbon electrodes, as listed in Table 3.

**Figure 7:**
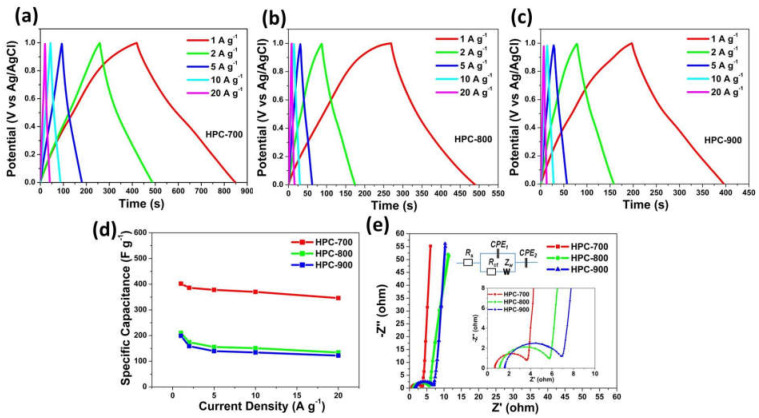
GCD curves of HPC-700 (**a**), HPC-800 (**b**), and HPC-900 (**c**) at different current densities; (**d**) the rate performance and (**e**) Nyquist plots of HPC-700, HPC-800, and HPC-900.

In addition, the high *V*_meso_ of the carbon samples can improve the rate performance limited by the slow diffusion-controlled reaction kinetics in the micropores of porous carbon. The CV curves of HPC-700 at scan rates from 10 to 100 mV s^−1^ are shown in Figure 6c. The maintained rectangular shapes combined with wide peaks around 0.2–0.6 V suggest the ideal capacitive behavior of HPC-700 at high scan rates. The specific capacitance of HPC-700 at a high scan rate is obviously higher than that of HPC-800 and HPC-900 according to the area of the CV curves (Figure 6d,e). As shown in Figure 7a–c, the GCD curves of the samples at the different current densities were measured to study their rate performance as the electrode materials of supercapacitors. Based on the GCD data, Figure 7d shows that HPC-700 supplies a capacitance of 346 F g^−1^ at 20.0 A g^−1^ with a capacitance retention of 86.1%, higher than the 134 F g^−1^ of HPC-800 with a capacitance retention of 63.6% and the 122 F g^−1^ of HPC-900 with a capacitance retention of 61.31% at 20 A g^−1^. The excellent rate performance of HPC-700 confirms that the high *V*_meso_ can provide the rapid transport pathways to enhance the diffusion kinetics of ions in the micropores of the carbon electrode at high charge/discharge rates effectively.

The ion transfer kinetics in carbon electrodes were further investigated by EIS. Figure 7e displays the Nyquist diagrams of the porous carbon electrodes; the inset is the corresponding equivalent circuit. The fast ion adsorption/desorption kinetics at the interfaces of the electrolyte/electrodes is proven by the steep lines almost perpendicular to the X-axis in the low-frequency region [48] owing to the very small diffusion polarization. The smaller diameter of the semicircle in the high-frequency region corresponds to the lower charge transfer resistance (*R*_ct_, 3.1 Ω) of HPC-700 than HPC-800 (*R*_ct_, 4.9 Ω) and HPC-900 (*R*_ct_, 5.2 Ω) [48]. In addition, the fast electrolyte ion diffusion process in the HPC-700 electrode is also verified by the lower Warburg resistance (*Z*_w_, 0.42 Ω) of HPC-700 than HPC-800 (*Z*_w_, 0.53 Ω) and HPC-900 (*Z*_w_, 0.58 Ω) in the middle-frequency region. The EIS results further indicate that the high *V*_meso_ of HPC-700 can reduce the ion transfer and diffusion resistance to accelerate the interface reaction kinetics in the carbon electrode. Therefore, a good balance between the high SSA and the rational *V*_meso_ enables the excellent capacitance and rate performance of HPC-700. 

**Figure 8:**
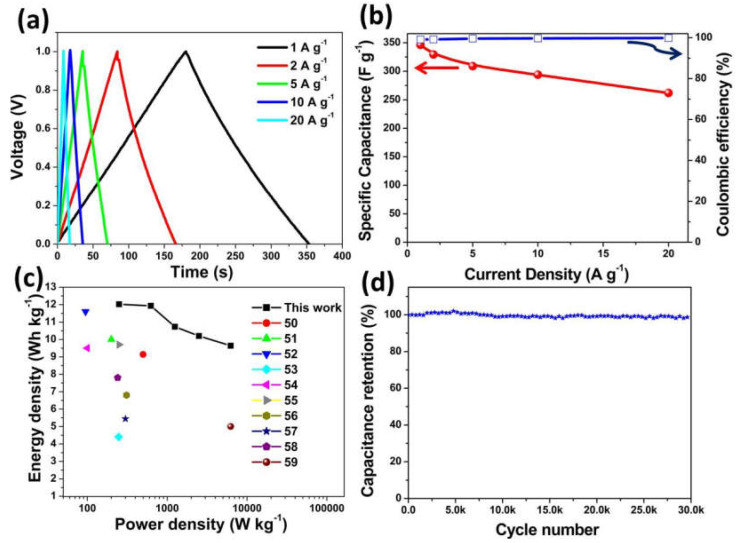
GCD curves (**a**) and specific capacitance, as well as Coulombic efficiency (**b**) of HPC700 at different current densities in HPC-700//HPC-700. (**c**) Ragone plot and (**d**) cycling stability of HPC700//HPC-700 at 5.0 A g^−1^ after 30,000 cycles.

The HPC-700//HPC-700 symmetric supercapacitor was further assembled. The GCD curves (Figure 8a) of the supercapacitor show highly symmetric triangles at current densities from 1.0 to 20.0 A g^−1^, indicating the highly reversible charge and discharge process. Figure 8b manifests that HPC-700 still shows a high specific capacitance of 346 F g^−1^ at 1.0 A g^−1^ and 262 F g^−1^ at 20.0 A g^−1^ in the supercapacitor, with a high Coulombic efficiency of 99.2%. The symmetric supercapacitor supplies an energy density of 12.02 Wh kg^−1^ at a power density of 250 W kg^−1^ (Figure 8c), which are higher than the results for bio-derived carbon-based supercapacitors reported in the literature [50,51,52,53,54,55,56,57,58,59]. Meanwhile, the supercapacitor also possesses a prolonged cycling life with 98.59% of the initial capacitance after 30,000 cycles at 5.0 A g^−1^ (Figure 8d). The excellent electrochemical performance of the supercapacitor using HPC-700 as the electrode material is due to the good balance of the high SSA and *V*_meso_ in HPC-700, which can make a large amount of the micropores accessible for charge storage through the expressways of the mesopores for ion transport. 

## 4. Conclusions

In conclusion, a good balance between a high SSA and *V*_meso_ in the biomass-derived porous carbon was achieved by using molten salt as a mesopore-generating agent during the precarbonization process and KOH as a further micropore-forming agent. The mesopore/micropore structure of the porous carbon can be tuned by changing the precarbonization temperatures. The mesopores generated by the molten salt in the precarbonized product can further serve as the active sites for the KOH activation to produce micropores. The high SSA and *V*_meso_ of the porous carbon provide a sufficient electrode/electrolyte interface to facilitate the electrolyte ion penetration and ion transfer expressways to accelerate the transport kinetics by diffusion control in the micropores. Consequently, the obtained carbon electrode exhibits a high specific gravimetric capacitance of 402 F g^−1^ at 1.0 A g^−1^ in a three-electrode system. In a symmetric supercapacitor, the obtained carbon delivers an excellent specific gravimetric capacitance of 346 F g^−1^ at 1.0 A g^−1^, as well as a 98.59% capacitance retention after 30,000 cycles at 5.0 A^−1^. This work has fundamental merits for enhancing the electrochemical performance of the biomass-derived porous carbon by optimizing the pore structures and SSA. 

## Figures and Tables

**Table 1:** Carbon yield of the different synthesis processes.

Samples	The Mass of Wheat Husk or Carbonized Product (g)	The Mass of Salt or KOH (g)	The Mass of Carbon Product (g)	Carbon Yield (wt%)
MHPC-700	4 (wheat husk)	16 (salt)	0.81	20.25
MHPC-800	4 (wheat husk)	16 (salt)	0.57	14.25
MHPC-900	4 (wheat husk)	16 (salt)	0.19	4.75
NHPC-700	2 (wheat husk)	0 (salt)	0.57	28.5
HPC-700	0.1 (carbon)	0.2 (KOH)	0.056	56
HPC-800	0.1 (carbon)	0.2 (KOH)	0.055	55
HPC-900	0.1 (carbon)	0.2 (KOH)	0.055	55

**Table 2:** Pore textural properties of the as-obtained porous carbon materials.

Samples	*S*_total_ (m^2^ g^−1^)	*V*_total_ (cm^3^ g^−1^)	*V*_micro_(cm^3^ g^−1^)	*V*_meso_ (cm^3^ g^−1^)
HPC-700	2721.	1.66	0.79	0.87
HPC-800	2202	1.41	0.75	0.65
HPC-900	1739	1.01	0.69	0.32
NHPC-700	149	0.11	0.03	0.08
MHPC-700	524	0.42	0.19	0.23
MHPC-800	361	0.28	0.15	0.14
MHPC-900	309	0.26	0.11	0.15

*S*_total_: total BET specific surface area; *V*_total_: total pore volume; *V*_micro_: micropore volume; *V*_meso_: mesopore volume.

**Table 3:** Specific capacitance of HPC-700 and some biomass-derived porous carbon reported in the literature.

Carbon Precursors	*C*_G_ (F g^−1^)	Electrolyte	Current Density (A g^−1^)	Refs.
Wheat husk	402	1 M H_2_SO_4_	1.0	This work
Sugarcane bagasse	371	1 M H_2_SO_4_	0.1	[38]
Sucrose	143	6 M KOH	1.0	[39]
Peach gum	426	1 M H_2_SO_4_	0.5	[40]
Flaxseed residue	369	6 M KOH	0.5	[41]
Rice husk	163	6 M KOH	0.2	[42]
Tobacco waste	197	1 M H_2_SO_4_	0.2	[43]
Grape marcs	446	1 M H_2_SO_4_	0.5	[44]
Mangifera indica peels	402	1 M H_2_SO_4_	1.0	[45]
Dead plant leaves	345	1 M H_2_SO_4_	0.5	[46]
Foxtail grass seeds	358	6 M KOH	0.5	[47]
Egg white	304	1 M H_2_SO_4_	1.0	[48]
Pomelo peel	314	6 M KOH	1.0	[49]

## Data Availability

The data presented in this study are available upon request from the corresponding author. The data are not publicly available due to privacy. Data are contained within the article.
